# Deep mining in the dorsal raphe: cholecystokinin-expressing neurons encode satiation-related cues to regulate meal size

**DOI:** 10.1038/s41392-025-02240-9

**Published:** 2025-05-16

**Authors:** Niels Röhrdanz, Peer Wulff, Kira Balueva

**Affiliations:** Institute of Physiology, Christian-Albrechts-University Kiel, Kiel, Germany

**Keywords:** Cellular neuroscience, Molecular neuroscience

In a recent article published in *Cell*,^1^ Chowdhury and colleagues apply advanced single-cell mRNA profiling to identify a previously unknown strictly peptidergic neuronal population in the dorsal raphe nucleus (DRN). Prompted by pathway-enriched genes, the authors show that these cholecystokinin (CCK)-expressing neurons integrate a broad spectrum of food intake- and satiation-related signals and play a robust role in regulating meal sizes during homoeostatic eating (Fig. 1).

**Fig. 1:**
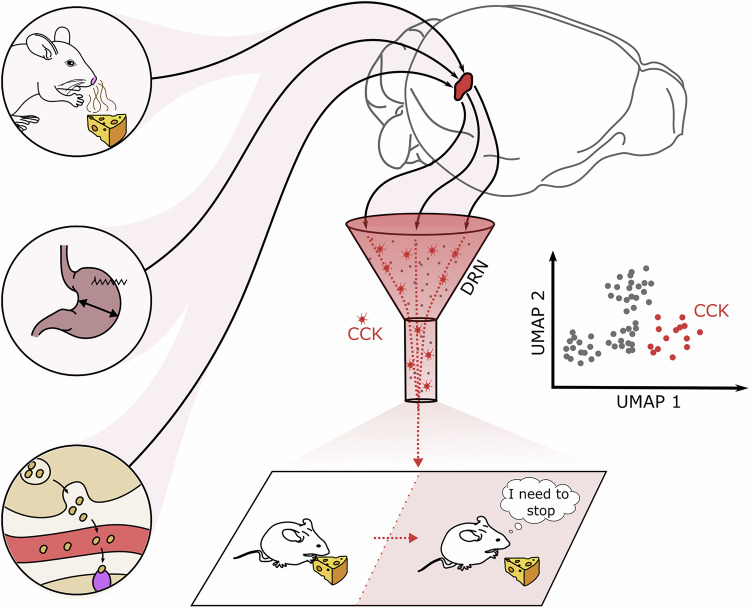
Cholecystokinin-expressing neurons respond to satiation-related cues to control meal size. CCK-neurons in the dorsal raphe nucleus respond to the mere presence of food, mechano- and chemosensory cues from the gut, as well as humoral factors that are released in relation to ingestion. These neurons were identified in a spatial transcriptomic experiment as obligatory peptidergic. Activation of CCK+ neurons leads to a significant decrease in meal size

Like body temperature or water balance, energy balance is homeostatically regulated via feedback loops, which allow us to precisely balance energy intake and expenditure. A positive energy balance, that is, we eat more than we need, may result from faulty feedback and lead to obesity. What are the feedback circuits that regulate food intake? Hedonic eating is largely motivated by reward association and relies on the mesolimbic dopaminergic system. In contrast, homoeostatic control of eating uses humoral and neuronal signals that relay information about peripheral energy levels and food uptake to the hypothalamus and brainstem. While much progress has been made regarding the hypothalamic circuits that sense body energy stores via leptin and insulin to generate hunger or satiety, less is known about shorter-acting mechanisms presumably located in the brainstem that control satiation during a meal.^2,3^ One candidate node of the respective neuronal circuitry is the DRN of the midbrain.

To deduce molecular and spatial organisation of the heterogeneous cell populations in the DRN, Chowdhury and colleagues apply two cutting-edge hydrogel-based spatially resolved single-cell transcriptomic and translatomic techniques: the large-scale spatially resolved transcript amplicon readout mapping (STARmap) and the ribosome-bound mRNA mapping technique (RIBOmap).^4^ Integrating these with previous single-cell data, they map out the cell types of the DRN to then zoom into a specific previously unrecognised cluster of cells expressing CCK, a neuropeptide previously implicated in eating behaviour. Diving deeper into the molecular make-up of these CCK+ neurons, they cell type selectively isolate actively translated mRNAs (a method termed vTRAP).^5^ vTRAP revealed that DRN CCK-neurons do not have fast neurotransmission and are neither dopaminergic nor serotonergic but likely obligatory peptidergic, expressing additional neuropeptides implicated in the regulation of food intake. Additionally, gene ontology analysis showed enrichment of genes involved in energy homoeostasis and nutrient sensing.

Following this transcriptome-derived lead, the authors assessed CCK neuronal activity during food intake. c-Fos immunohistochemistry showed CCK-neuron activation only in fasted mice that were refed but not in fasted or fed ad libitum subjects. In addition, mice scalably decreased food intake during artificial optogenetic stimulation of CCK-neurons. Chowdhury et al. concluded that CCK-neurons are activated by homoeostatic eating and then suppress food intake.

Using the genetically-encoded calcium indicator GCaMP6s and fibre photometry, the authors then investigated what exactly CCK-neurons encode. To improve analytical resolution, fasted mice were now refed in an automated system, which allows tracking of individual bites (single pellets) within a meal (defined as the consumption of several bites with no more than 60 s between bites). CCK-neuron activity increased with each pellet retrieval and returned to baseline within about 20–30 s. CCK-neurons thus encode individual bites and potentially meal size through temporal integration of activity.

Although the authors do not investigate the actual sensory stimulus that caused activation of CCK-neurons during food retrieval, they report that already sensory food perception (e.g., olfaction) increased neuronal activity. Importantly, besides exteroceptive cues, CCK + DRN neurons also responded to interoceptive cues. For example, injection of the orexigenic hormone ghrelin, released by the intestine during hunger, induced rapid silencing of CCK-neurons. In addition, intragastric infusion of nutritive and non-nutritive solutions showed slow onset activation of CCK + DRN neurons, suggesting responsiveness to mechano- and chemosensory cues of the gut, presumably transmitted by vagal afferents. Similarly, several gut-derived factors involved in satiation-signalling, such as oxytocin, amylin, CCK, and Glucagon-like peptide-1, activated CCK-neurons upon intraperitoneal injection. In contrast, CCK-neurons did not respond to cues such as pain or fear, which can suppress eating. CCK+ thus respond to a wide range of fast and slow-acting extero- and interoceptive cues related to energy balance. As the DRN is not a direct target of visceral afferents, and CCK-neurons do not express key receptors for the relevant humoral signals, most of the cues likely act via relays. Indeed, using rabies tracing, the authors detect several upstream relay sites previously implicated in eating control that do receive such visceral and/or humoral inputs (e.g., the nucleus tractus solitarii or the lateral parabrachial nucleus).

To probe the behavioural effects of CCK neuronal activation, Chowdhury and colleagues used optogenetics during refeeding. Activation effectively decreased meal sizes due to prolonged intervals between pellet retrievals. However, meal frequency was not affected. Activation of CCK-neurons just prior to food availability allowed to gauge the temporal dynamics of eating suppression, which started within minutes and lasted for about half an hour. How the activity of CCK-neurons is translated into behavioural action remains unknown. However, using virus-mediated tracing of axonal projections, the authors offer a number of possible downstream mediators, including several regions known to regulate feeding behaviour, such as the bed nucleus of the stria terminalis, the central extended amygdala or the lateral hypothalamus.

In summary, Chowdhury and colleagues effectively employed high-end molecular profiling to detect a new population of peptidergic CCK-expressing neurons in the DRN, which integrates external cues such as odour as well as numerous internal sensory as well as hormonal satiation-related cues to promote meal size reduction. Interesting questions arise from this study. What are the upstream pathways that provide satiation-related cues, and what are the downstream mediators of the behavioural effects? Are these downstream mediators indeed controlled strictly via peptidergic transmission? If this is so, these may not be limited to the axonal projection areas of CCK-neurons, as neuropeptide release is not restricted to synaptic terminals, and neuropeptides can exert effects over long distances. In addition, these neurons may release different neuropeptides in response to different inputs. Finally, how are DRN CCK-neurons tied into known circuits of energy balance and reward, and could these neurons be targeted to treat obesity?

## References

[1] Chowdhury, S. et al. Brainstem neuropeptidergic neurons link a neurohumoral axis to satiation. *Cell* **188**, 1563–1579.e1518 (2025). 39914383 10.1016/j.cell.2025.01.018

[2] Brüning, J. C. & Fenselau, H. Integrative neurocircuits that control metabolism and food intake. *Science* **381**, eabl7398 (2023). 37769095 10.1126/science.abl7398

[3] Alcantara, I. C., Tapia, A. P. M., Aponte, Y. & Krashes, M. J. Acts of appetite: neural circuits governing the appetitive, consummatory, and terminating phases of feeding. *Nat. Metab.* **4**, 836–847 (2022). 35879462 10.1038/s42255-022-00611-y PMC10852214

[4] Zeng, H. et al. Spatially resolved single-cell translatomics at molecular resolution. *Science* **380**, eadd3067 (2023). 37384709 10.1126/science.add3067 PMC11146668

[5] Nectow, A. R. et al. Rapid molecular profiling of defined cell types using viral TRAP. *Cell Rep.* **19**, 655–667 (2017). 28423326 10.1016/j.celrep.2017.03.048 PMC5476221

